# Minimally invasive cardiac surgery via bilateral thoracotomy in treatment of left ventricle aneurysm: a case report

**DOI:** 10.1186/s40792-023-01640-9

**Published:** 2023-04-13

**Authors:** Yuta Kanazawa, Shunsuke Saito, Ikuko Shibasaki, Taiki Matsuoka, Shotaro Hirota, Shouhei Yokoyama, Yasuyuki Kanno, Masahiro Tezuka, Go Tsuchiya, Taisuke Konishi, Koji Ogata, Hirotsugu Fukuda

**Affiliations:** Department of Cardiac and Vascular Surgery, Dokkyo Medical University, 880 Kitakobayashi, Mibu, Tochigi 321-0293 Japan

**Keywords:** Minimally invasive cardiac surgery, Left ventricle aneurysm

## Abstract

**Background:**

Left ventricle aneurysm (LVA) as a sequela to myocardial infarction or iatrogenic injury is required surgical treatment with full median sternotomy. Herein, we report a case of successful surgical treatment of left ventricle aneurysm performed by minimally invasive cardiac surgery (MICS).

**Case presentation:**

We describe a case of a LVA treated by minimally invasive cardiac surgery in an 82-year-old woman who reported to the hospital with the complaint of chest pains at rest. Computed tomography (CT) coronary angiography revealed a left ventricle apical aneurysm. The aneurysm was suspected to be a pseudoaneurysm caused by a previous myocardial infarction. Surgery was performed under general anesthesia, with the patient in a supine position. A small incision was made in the 3rd intercostal space through which an aortic root vent cannula and aortic clamp were inserted, followed by exposing the aneurysm via incision of the left 6th intercostal space. The aneurysm was resected and pathologically examined, revealing it to be a “true” aneurysm. The left ventricle wall was closed using polypropene mattress sutures. Postoperative CT scan revealed successful resection of the aneurysm. Usually, a surgical treatment with full median sternotomy and left ventriculostomy is indicated for LVA. We decided to treat the LVA with bilateral thoracotomy MICS. We preferred to perform this procedure under cardiac arrest to ensure safe and secure closure of the aneurysm. The right small thoracotomy was necessary for aortic cross-clamping and aortic root venting.

**Conclusions:**

The procedure was safe and simple and yielded excellent postoperative outcomes. Therefore, we speculate that this method can be applied to the management of larger aneurysms.

## Background

Aneurysm of the left ventricle that arises as a sequela to myocardial infarction or iatrogenic injury. An idiopathic left ventricle aneurysm is rare. Typically, a surgical treatment with full median sternotomy is required for the management of this lesion. Herein, we report a case of successful surgical treatment of left ventricle aneurysm performed by minimally invasive cardiac surgery (MICS).

## Case presentation

**Fig. 1:**
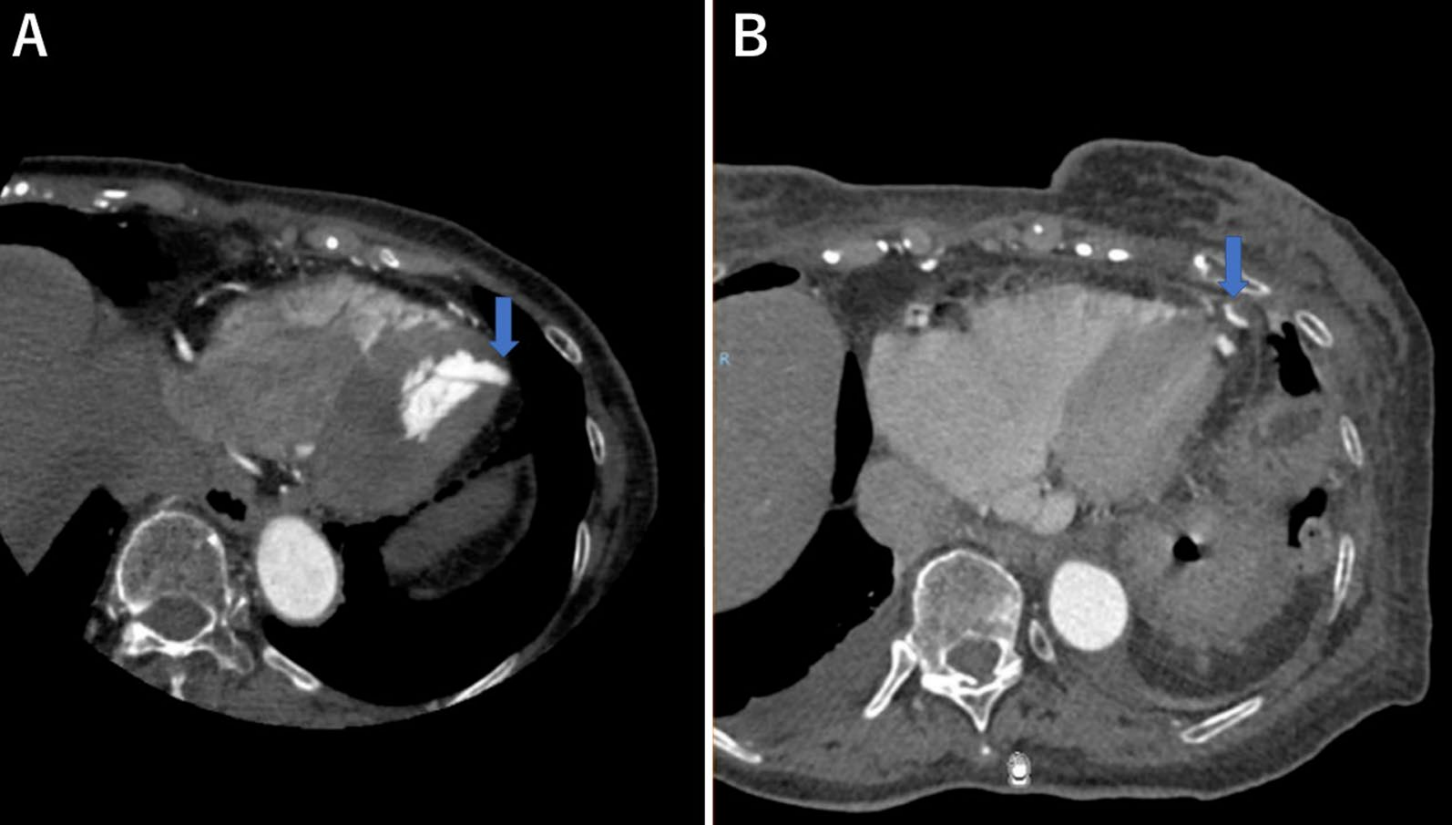
Pre- and postoperative CT scan images. **a** Preoperative CT scan images. Allows: Left ventricle aneurysm. **b** Postoperative CT scan images. *CT* computed tomography

An 82-year-old female patient with a history of hypertension visited a local hospital after experiencing chest pains at rest during the night. As a result of suspected angina, coronary computed tomography (CT) angiography was performed, revealing an intact coronary artery; however, an apical aneurysm of the left ventricle was detected (Fig. 1a). Transthoracic echocardiography showed dyskinetic wall motion consistent with the left ventricle aneurysm. Based on the CT findings and clinical history of the patient, a vasospastic angina was suspected. The patient was started on a calcium-channel blocker, which relieved the symptoms effectively. The left ventricle apical aneurysm was caused by a previous myocardial infarction due to vasospastic angina. Therefore, we planned to conduct a surgical intervention for the aneurysm via MICS procedure.

**Fig. 2:**
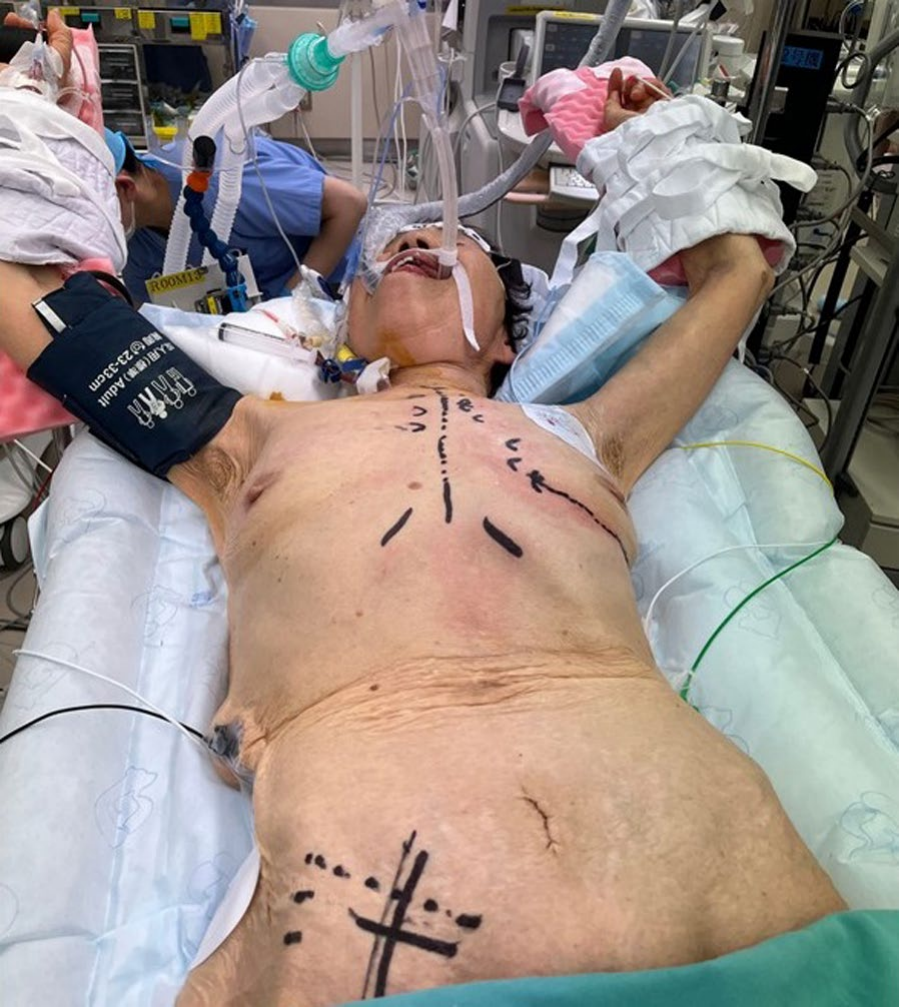
Operative position. The patient was placed in the supine position with both arms raised

**Fig. 3:**
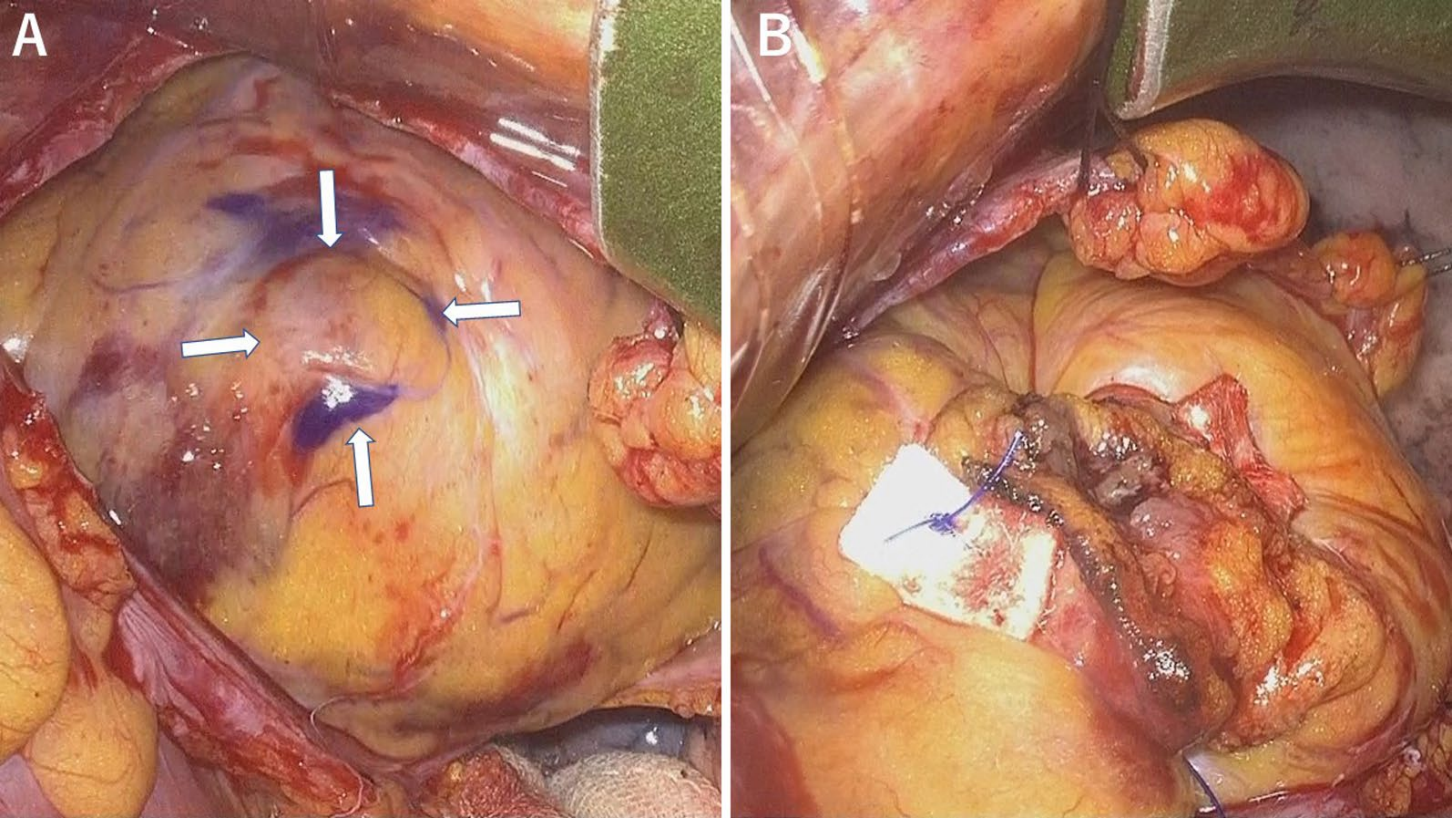
Intraoperative images. **a** Left ventricle aneurysm at apex: Allows. **b** Resected and sutured aneurysm

The patient was placed in a supine position with both arms raised (Fig. 2). General anesthesia was induced by a standard technique with a double-lumen tube for unilateral ventilation. Peripheral extracorporeal circulation was established via the right femoral artery and vein. An aortic root vent cannula and aortic clamp were inserted via the right 3rd intercostal space after creating a small incision. The aneurysm was exposed via a small incision of the left 6^th^ intercostal space. When the left ventricle was filled with blood, an area of dyskinetic movement was detected, which was confirmed as an aneurysm on epicardial echocardiography. There was no adhesion or scarring from the old myocardial infarction. The aneurysm was 1.5 cm × 1.5 cm (Fig. 3a) in size. Cardiac arrest was achieved by administrating antegrade cardioplegia, and the aneurysm was resected. The left ventricle wall was closed using felt-pledgetted 3–0 polypropylene mattress suture (Fig. 3b). Operation time was 184 min.

The postoperative course was uneventful without any complications, and she was discharged after 14 days. Postoperative CT images revealed successful resection of the left ventricle aneurysm (LVA) (Fig. 1b). Pathological examination demonstrated that the cardiac muscle had little fibrotic and hypertrophic changes, and there was no evidence of secondary cardiomyopathy and cardiac infarction. Finally, the aneurysm was diagnosed as an idiopathic LVA.

## Discussion

An LVA is an infrequent (5–10%) complication of myocardial infarction [1]. Other reported potential etiologies of LVAs are trauma, hypertrophic cardiomyopathy, cardiac sarcoidosis, and more exotic causes such as Chagas disease [2]. Cases of idiopathic LVA have also been reported, but the exact incidence is unknown. An idiopathic LVA is diagnosed by ruling out all possible etiologies of LVA based on the patient’s medical history, clinical examination, and laboratory test results [3, 4].

Usually, a surgical treatment with full median sternotomy and left ventriculostomy is indicated for LVA. Alternatively, closure via the left atrium and percutaneous closure have also been reported [5, 6]. However, to date, there has been no report on LVA closure with lateral thoracotomy MICS.

In this case, we decided to treat the LVA with bilateral thoracotomy MICS. Theoretically, “off-pump” closure or “on-pump beating” closure of the LVA was possible in the present case, which would have spared the right thoracotomy. However, we preferred to perform this procedure under cardiac arrest to ensure safe and secure closure of the aneurysm. Moreover, to achieve complete closure of the aneurysm at its neck, we considered it essential to get a clear view inside the aneurysm by adequate drainage of blood from the heart, and therefore root vent cannula was indispensable in order to prevent air embolism during cardiopulmonary bypass support. The right small thoracotomy was necessary for aortic cross-clamping and aortic root venting. Preoperative CT scan and transthoracic echocardiogram results obtained with the patient in the surgical position proved to be exceedingly useful for the selection of the left thoracotomy height. The left thoracotomy made it possible to approach the cardiac apex with ease, and a good field of view of the aneurysm was obtained. We believe that this technique has the potential to be applied to left ventriculoplasty for treatment of larger aneurysms. However, this technique can’t be applied if concomitant coronary artery bypass grafting is required.

## Conclusions

We performed a successful operation for repair of LVA by MICS via bilateral thoracotomy. The procedure was safe and simple and yielded excellent postoperative outcomes. Therefore, we speculate that this method can be applied to the management of larger aneurysms.

## Data Availability

Not applicable.

## Abbreviations

LVA: Left ventricular aneurysm
MICS: Minimally invasive cardiac surgery
